# Automated design of freeform imaging systems

**DOI:** 10.1038/lsa.2017.81

**Published:** 2017-10-06

**Authors:** Tong Yang, Guo-Fan Jin, Jun Zhu

**Affiliations:** 1 State Key Laboratory of Precision Measurement Technology and Instruments, Department of Precision Instrument, Tsinghua University, Beijing 100084, China

**Keywords:** automated design, construction and iteration, freeform imaging system, point-by-point

## Abstract

The automated design of imaging systems involving no or minimal human effort has always been the expectation of scientists, researchers and optical engineers. In addition, it is challenging to choose an appropriate starting point for an optical system design. In this paper, we present a novel design framework based on a point-by-point design process that can automatically obtain high-performance freeform systems. This framework only requires a combination of planes as the input based on the configuration requirements or the prior knowledge of designers. This point-by-point design framework is different from the decades-long tradition of optimizing surface coefficients. Compared with the traditional design method, whereby the selection of the starting point and the optimization process are independent of each other and require extensive amount of human effort, there are no obvious differences between these two processes in our design framework, and the entire design process is mostly automated. This automated design process significantly reduces the amount of human effort required and does not rely on advanced design skills and experience. To demonstrate the feasibility of the proposed design framework, we successfully designed two high-performance systems as examples. This point-by-point design framework opens up new possibilities for automated optical design and can be used to develop automated optical design in the areas of remote sensing, telescopy, microscopy, spectroscopy, virtual reality and augmented reality.

## Introduction

Optical design has been a topic of considerable importance in advancing how humans explore the unknown world. In the past, optical design was tedious ‘manual’ work. Now, with the rapid development in computer science and technologies, the general method for performing an optical design task is first to find a starting point originating from patents or other existing systems and then to use computer-aided optimization. For example, the standard or global optimization in optical design software such as Code V is a powerful tool for realizing optical design with proper boundary conditions and a proper starting point. However, because off-axis or other special configurations are increasingly being used, viable starting points for specific design forms are generally limited^1^. Consequently, an extensive amount of human effort is needed to find proper starting points. Furthermore, the optimization process requires a ‘mental and technical equilibrium with the task at hand’^2^ because it highly relies on the guidance, experience, and skill of designers. When designing systems with advanced specifications and/or special system configurations, feasible starting points are rare or cannot be found. If the specifications, configurations and number of elements of the starting points are far from the prescribed design, extensive optimization is essential. Sometimes, designers may even fail to achieve a satisfactory solution.

In recent years, developments in advanced manufacturing technologies have resulted in freeform surfaces being successfully applied to many imaging fields^3, 4^, such as unobscured off-axis reflective systems design^5, 6, 7, 8, 9^, head-mounted-displays^10, 11, 12, 13, 14, 15^, freeform microlens arrays^16^, and panoramic optical systems^17^. However, it is harder to design a high-performance freeform optical system using the traditional design method. To avoid the extensive amount of human effort needed to find starting points and the subsequent optimization process, we propose a novel design framework whereby automated freeform optical design is realized via a point-by-point design process that only requires a combination of planes as the input.

Compared with the traditional design method, whereby the selection of the starting point and the optimization process are independent of each other and both require extensive amount of human effort, there are no obvious differences between these two processes in our design, and the entire design process is mostly automated. The designers only need to specify the initial planar system, based on the configuration requirements or their prior knowledge, as well as the desired object-image relationships and other particular constraints used in the design. This framework realizes the design starting from simple planes; thus, the dependence on existing starting points is significantly reduced. This approach is different from traditional design, which starts from a starting point with a certain optical power. In addition, the automated design process significantly reduces the amount of human effort required and does not rely on advanced design skills and experience. This point-by-point design framework opens up new possibilities for automated optical design and can be applied in many areas, such as remote sensing, high-performance telescopy, microscopy, spectroscopy, VR and AR. To demonstrate the feasibility of the proposed idea, we present two design examples that exhibit high performance.

## Materials and methods

### Preparing the design inputs

In our design framework, an initial planar system along with the desired object-image relationships and other particular constraints of the system need to be specified before conducting the automated design process. The basic configuration or folding geometry of the system is determined before the design process begins and is based on the design requirements or the prior knowledge of the designers. The decentered and tilted planes in the initial system should be located in approximately the same places as the final freeform surfaces. In addition, the initial planes should redirect the light rays in a manner similar to that of the final freeform system, and the obscuration can be eliminated. For example, if we want to design a freeform, off-axis, three-mirror system using a Wetherell configuration or Cook configuration, the three initial planes should be located in approximately the same places as the final freeform surfaces. If we want to achieve a compact and special configuration in which the light beams are overlaid, the initial planes should be located such that they redirect the light rays in approximately the desired way. In conclusion, the locations of the initial planes may not be exactly the same as those of the final design but should not be too far from the expected locations. In addition, the initial planes should form the same type of folding geometry required. The proposed method is not able to find solutions that have a folding geometry different from that of the initial system. The folding geometry of the system is determined before beginning the design process. During the design process, the folding geometry type is maintained by using structure constraints, which means that the folding geometry type of the final design will not change. In addition, if the starting geometries of the initial systems within the same type of system configuration or with the same folding geometry are similar, the final design results will be similar. If the starting geometries of the initial systems are markedly different, the final designs will fall into other local optimums. The stop location is determined before commencing the design process, and it is not a degree of freedom during the design process.

Multiple feature rays should be employed which come from different pupil coordinates and different fields, as it is a general case of designing non-rotationally symmetric freeform surfaces. For example, polar ray grid can be used to define the feature rays in conventional optical systems using circular apertures^18^. For each field, we divide the aperture into *N* angles of different polar directions and we pick *P* pupil coordinates along each polar direction. If the total number of fields during the design is *M*, the total ray number *K* is *N* × *P* × *M*. Other methods for choosing different rays among full field-of-view (FOV) and aperture can also be used. The imaging system design approach can be considered as redirecting the multiple feature light rays to their target points on the image plane. The feature data points corresponding to each surface are obtained based on this goal. These points can be considered as the points where the rays intersect with the freeform surface.

**Figure 1:**
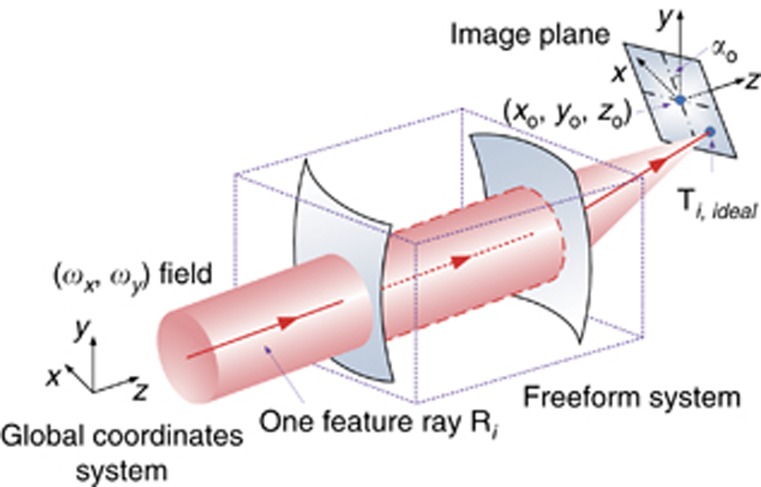
Schematic view of the ideal target point T_*i,ideal*_ for the rays from a specific sample field (*ω*_*x*_, *ω*_*y*_) on the image plane.

The ideal target points (the ideal image points in the general case) **T**_***i,ideal***_ for each feature ray **R**_***i***_ (*i*=1,2…*K*) on the ideal image plane can be calculated on the basis of the given object-image relationships and image plane position. Here, we consider the conventional case whereby the optical system has plane symmetry about the meridional plane. As shown in Figure 1, for a feature ray from a specific sample field (*ω*_*x*_, *ω*_*y*_), the global coordinates of **T**_***i,ideal***_ can be written as


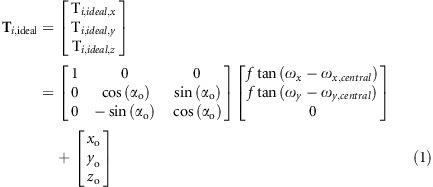


where *f* represents the focal length for the system; (*x*_o_,*y*_o_*,z*_o_) are the global coordinates of the center of the image plane (the ideal image point of the central field (*ω*_*x*,*central*_, *ω*_*y*,*central*_) among the full FOV); *α*_o_ is the tilt angle of image plane relative to the global *x*-axis. When the image plane position changes during the design process, **T**_***i,ideal***_ has to be recalculated. Sometimes **T**_***i***_≠**T**_***i,ideal***_ in a special case, which will be discussed later.

During the optical design process, proper constraints are essential to define boundary conditions that control the optical or physical properties^2^. Here, the object–image relationships are primarily constrained by controlling the target points of the feature light rays using the point-by-point freeform surface design process, described in the following sections. Therefore, the constraints are mainly used to eliminate light obscuration, avoid surface interference and achieve the required system configuration.

### Framework of the freeform surface design process

We propose a novel framework whereby we can use a point-by-point design process to realize automated freeform systems design. This approach is different from the traditional design method based on the software optimization of surface coefficients, whereby the selection of the starting point and the optimization process are independent of each other and require extensive amounts of human effort. In our framework, there are no obvious differences between these two processes in our design, and the entire design process is mostly automated. Traditional software optimization of the surface coefficients is no longer needed, and this design framework provides an alternative and promising way to design freeform surfaces and systems. The design of the freeform surfaces comprises two aspects. One aspect involves determining the shapes of the freeform surfaces, which includes two key techniques: the calculation of feature data points and freeform surface fitting. The other aspect involves determining the locations of the freeform surfaces.

### Determining the shapes of the freeform surfaces

The freeform surface shapes are generated via a point-by-point design process. A key technique for determining the freeform surface shapes is how to obtain the feature data points. For the imaging systems design, a basic requirement for the calculation of the feature data points is taking rays from different pupil coordinates and full fields into account. Among the existing point-by-point design methods^19, 20, 21, 22, 23, 24, 25, 26, 27^, the Construction–Iteration (CI) process^27^ satisfies the above requirements and is a good choice for the freeform surface shape design. Other potential point-by-point design approaches satisfying the above requirements can also be used.

The other key technique for determining the shapes of the freeform surfaces is the fitting of the freeform surfaces. There are many ways to realize the freeform surfaces fitting. Traditional fitting methods consider only the coordinates of the points. However, the propagation of rays is not only sensitive to coordinates of the feature data points, but also sensitive to the surface normals according to Snell’s Law (or the Law of reflection). If the surface normals are not considered during surface fitting, the surface normals after fitting may have large deviations from the prescribed directions. Consequently, the optical performance will not reach the expectations. One way to solve this problem is to use large numbers of data points during the traditional surface fitting. However, the total number of rays should not be too large to avoid a long computation time. Therefore, in our design process, it is recommended that surface fitting process should considers not only the coordinates of the feature data points, but also their surface normals^28, 29^. To obtain freeform polynomial surfaces, for example, XY polynomials or Zernike polynomials, we can use the method presented in Ref. 28. If splines or NURBS are required in other applications, the method given in Ref. 29 or other potential fitting methods can be used.

**Figure 2:**
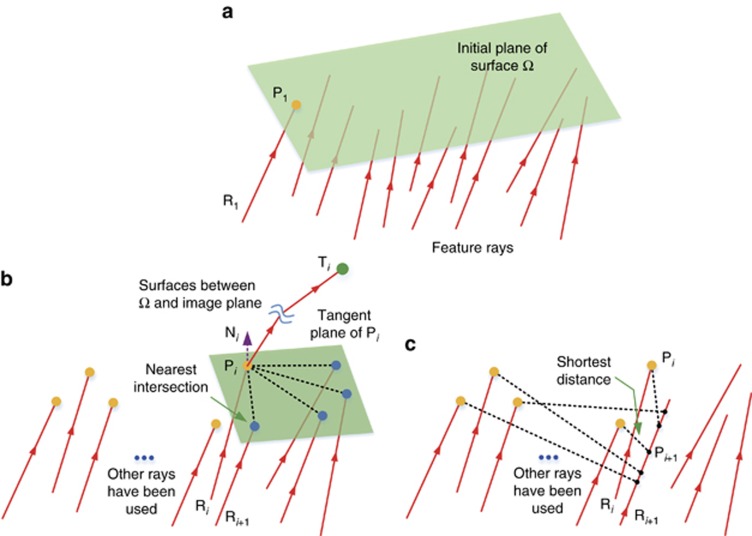
Method for calculating the feature data points of surface Ω in the preliminary construction stage. Only one surface in the system is shown here. (**a**) Calculation of the initial data point P_1_. (**b**) Determining the feature ray R_*i*+1_, when P_*i*_ is obtained. (**c**) Determining P_*i*+1_. The data points which have been obtained are painted in yellow.

**Figure 3:**
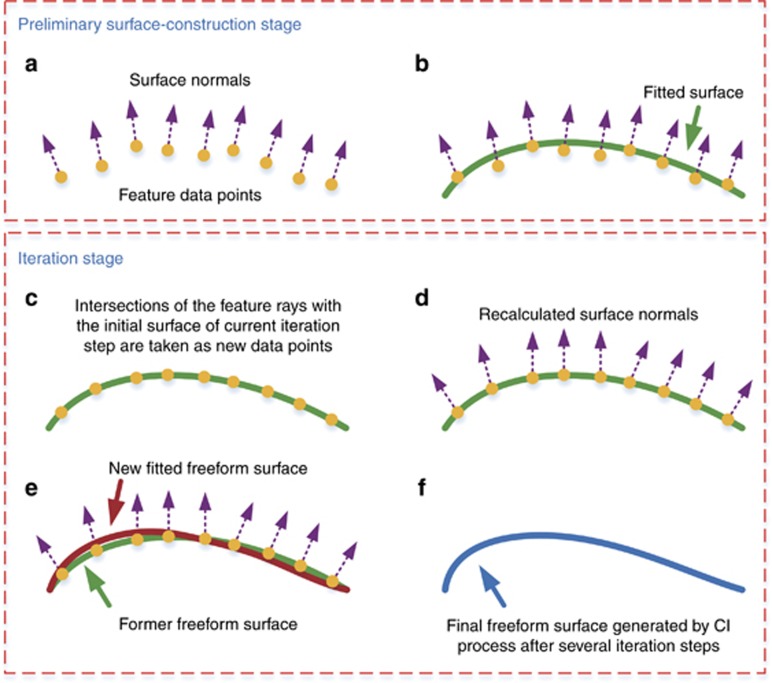
2D schematic of the entire CI process. (**a**,**b**) show the preliminary construction stage, and (**c**–**f**) show the iteration stage. Only one surface in the system is shown here. **a** Calculation of the data points. **b** Surface fitting in the construction stage. **c** Retaining of the intersection points of the feature rays with the initial surface as the new feature data points. **d** Recalculation of the surface normals of feature data points. **e** Surface fitting in one step of the iteration process. **f** Final freeform surface designed by using the CI process.

Next, we present some details of the freeform surface shape design using the CI process, which has two stages. The first stage is the preliminary surface-construction stage. The second stage is the iteration stage. In both stages, the freeform surfaces are always designed one by one. In the preliminary surface–construction stage, each unknown freeform surface **Ω** in the system is constructed using the feature data points **P**_***i***_ (*i*=1,2...*K*) corresponding to the feature light rays **R**_***i***_ (*i*=1,2…*K*). First, we find the point where the first ray **R**_**1**_ intersects with the initial planar surface of **Ω**, as shown in Figure 2a. This point is employed as the first data point **P**_**1**_. The remainder of the data points are obtained on the basis of the ‘nearest-ray algorithm’^18^. When we have calculated the *i*th (1≤*i*≤*K*−1) data point **P**_***i***_, we hope that this point can redirect the corresponding feature ray **R**_***i***_ to its corresponding target point **T**_***i***_ (generally, the ideal image point **T**_***i,ideal***_). The variation of the optical path length between **P**_***i***_ and **T**_***i***_ is zero based on Fermat’s principle. Therefore, the ray path between **P**_***i***_ and **T**_***i***_ is determined, and the outgoing direction of **R**_***i***_ at **P**_***i***_ can be calculated. In addition, the incident direction of **R**_***i***_ at **P**_***i***_ is known. Then, based on Snell’s Law, we can obtain the surface normal **N**_***i***_, as well as the tangent plane at **P**_***i***_, as shown in Figure 2b. Next, the *K*−*i* points are calculated where the rest of feature rays intersect with the tangent plane at **P**_***i***_. The nearest intersection to **P**_***i***_ is then determined. The corresponding feature ray is defined as the next feature ray **R**_***i*****+1**_. However, **P**_***i***_ may not be the nearest data point to **R**_***i*****+1**_. Then, the *i* intersections, that is, where **R**_***i*****+1**_ intersects with the tangent planes of the existing *i* data points, are calculated. The ‘data point-intersection’ pair that has the minimum distance between two points is obtained, as shown in Figure 2c. The corresponding intersection of this pair is the next data point **P**_***i*****+1**_. Then we can calculate the normal **N**_***i*****+1**_ at **P**_***i*****+1**_. The above steps are repeated until all *K* data points on **Ω** are calculated (depicted in Figure 3a). Then, through surface fitting, we can obtain the freeform surface, as shown in Figure 3b.

After the above process, the first unknown freeform surface **Ω** is generated, and the initial plane can be replaced. All the unknown freeform surfaces can be generated successively using this process, and the preliminary surface construction stage is completed. But the actual image points of the feature rays may be far from the ideal image points. Therefore, the iteration process is used to regenerate the freeform surfaces and reduce the deviations^27^. We take the system obtained after completing the preliminary construction process as the new initial system. When regenerating each surface in each iteration step, the coordinates of the points where the rays intersect with the initial surface are preserved as the coordinates of new feature data points, as shown in Figure 3c. The target points for the rays during iterations are generally their corresponding ideal image points. To accelerate the iteration process, an alternative negative feedback mode can be used. Here, the target point **T**_***i***_ for each ray is determined on the basis of its ideal target point **T**_***i,ideal***_ (also the ideal image point) as well as its actual image point **T**_i_^*^ before the current surface is regenerated: **T**_***i***_=**T**_***i,ideal***_+*ε*(**T**_***i,ideal***_−**T**_i_^*^), *ε*>0. Then, the surface normals of these points are recalculated based on Snell’s Law and Fermat’s principle, as shown in Figure 3d. Then, the new surface can be regenerated through surface fitting, as shown in Figure 3e. With this method, the freeform surfaces are regenerated successively in each iteration step. The iteration step can be repeated to improve the optical performance and the final freeform surfaces designed by the iteration process can be obtained, as shown in Figure 3f. We can employ the root-mean-square (RMS) deviation *σ*_RMS_ of between the ideal and actual image points after each iteration step to show the effect of the design process. This is defined as


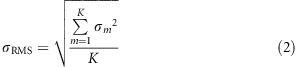


where *σ*_*m*_ is the deviation for the *m*th feature ray. More detailed discussions of the CI process can be found in Ref. 27.

### Determining the locations of the freeform surfaces

The process depicted above can be performed to obtain a freeform system in which the feature light rays are approximately redirected to the image points. However, this system is generally not ready for imaging at this step because the image quality may be far from the diffraction limit. In fact, the single CI process depicted above mainly controls the surface shape. The obtained system is a local optimum of the system if the surfaces are located at the current positions with certain tilts. Therefore, the positions and tilts of the surfaces and the image plane also need to be altered to improve the image quality. Many search or optimization methods can be used to change the positions and tilts of the freeform surfaces. Here, we use a single-freedom-search (SFS) strategy to achieve this goal, which will be discussed later in detail.

The position and tilt of one surface in the system can be characterized by its (*x*, *y*, *z*) positions in space and its tilt angles. If we predefine a global base coordinate system, the surface position can be defined by the *x*-decenter, *y*-decenter and *z*-decenter of the surface vertex with respect to the origin of the base coordinate system. The tilt of the surface can be defined by its *α*-tilt, *β*-tilt and *γ*-tilt (rotation angles about the global *x*-axis, *y*-axis and *z*-axis, respectively). Each of these parameters can be seen as a degree of freedom (DOF) in the optical design. For traditional systems that are symmetric about the *yOz* plane, the *y*-decenter, *z*-decenter and *α*-tilt of each surface (and the image plane) can adequately describe the surface position and tilt.

Here, we use the SFS strategy to improve the performance of the system. The system obtained by conducting the first CI process is taken as the first base system (BaseS#1). The total number of DOFs in the system used in the SFS process is called *TDOF* here. We select the first degree of freedom (DOF#1) for surface positions and tilts. DOF#1 is set to different discrete values within a reasonable range around the current value in the base system. In this way, a series of new ‘perturbed’ optical systems are obtained. Note that if a system with a new DOF value would definitely violate or strongly tend to violate the predefined constraints, this value (or the ‘perturbed’ system) should be eliminated. Then, the CI process (or just the iteration process) is repeated for each perturbed optical system to improve the image quality. If only the iteration process is employed, no feedback should be used (*ε*=0) for the first step of iterations to prevent instability^27^. The system with the minimum *σ*_RMS_ is taken as the best solution. If the minimum *σ*_RMS_ is smaller than that of the current base system, we take this best solution as the next base system (BaseS#2) for the search step of the next degree of freedom (DOF#2). Otherwise, the current base system (BaseS#1) is taken as the base system (BaseS#2) for DOF#2.

A similar process is used for the remaining DOFs and all the DOFs are used once from search step 1 to step *TDOF*. However, the design result after step *TDOF* may not be good. Therefore, the above search process can be repeated using the same DOFs for another round to achieve a satisfactory result starting from BaseS#(*TDOF*+1), which means that the same DOFs are used again from search step *TDOF*+1 to step 2 × *TDOF*. The sequence of different DOFs used in the SFS process and the discrete values for each DOF are predefined by the designer before beginning the automated design process. It is recommended that the optical surfaces are perturbed in the same sequence as that of their locations in the system.

The constraints predefined during setup are used in the SFS process. During the iterations for each perturbed system, if the constraints are violated, the iteration process for this perturbed system will be terminated immediately. This system will not be involved in finding the best solution for the current DOF. The constraints are loose at the beginning to achieve fast convergence of the image quality as well as of the distortion, and the constraints will become more stringent during the design process to generate satisfactory systems.

In addition, during the later stage of the automated design process, we can improve the performance of the system by sampling more feature rays in the CI process and/or by using a higher-order freeform surface (for example, higher-order XY polynomials or Zernike polynomials) during the surface fitting process. Moreover, in the early stage of the design process, the shapes of the surfaces may change significantly. This change in the surface shape may have an impact on the position and size of the entrance pupil^30^, thus changing the light rays used for imaging. It is recommended to use a larger entrance pupil size during the early stage of the automated design process to consider a wider range of feature rays. This process significantly reduces the effect of the surface shape variation. During the later stage of the design, the changes in the surface shape are small, and the actual entrance pupil size should be used.

In some design cases, there are strict requirements regarding the positions or tilts of the surfaces in the system. When designing these systems, these requirements should be satisfied when building initial planar system. In addition, the DOFs related to these requirements should only be changed slightly or should not be chosen for the SFS process. However, the positions or tilts of the surfaces may still slightly depart from the requirements at the end of the design process. At this time, the surfaces in the design result can be adjusted to completely satisfy the requirements, and an additional construction-iteration process (or only the iteration process) can be used to obtain the final result. Because it changes the position or tilt of the surfaces, this process can be considered as an additional search step.

### Optional high priority for image quality iteration mode

The goal of the traditional CI process is redirecting the feature rays to their ideal image points. The strength of this mode is that it controls both the image quality and distortion. During the early stage of the design process when the image quality is poor and the distortion is large, this mode is very effective because we want the feature light rays to quickly converge to the ideal image points. However, in the later stage of the design, especially when the system is close to high performance, this mode may be too strict. In this mode, the CI process will generate a freeform system that has the minimum *σ*_RMS_. This is a tradeoff between the image quality and distortion, but it does not necessarily yield the optimum image quality. In fact, the distortion is very small in the later stage of the design process and is generally within its allowed range. Additionally, the distortion can be calibrated and corrected by using other methods. Therefore, a better design strategy in the later stage of design is to impose stronger controls on the image quality and less control on the distortion.

**Figure 4:**
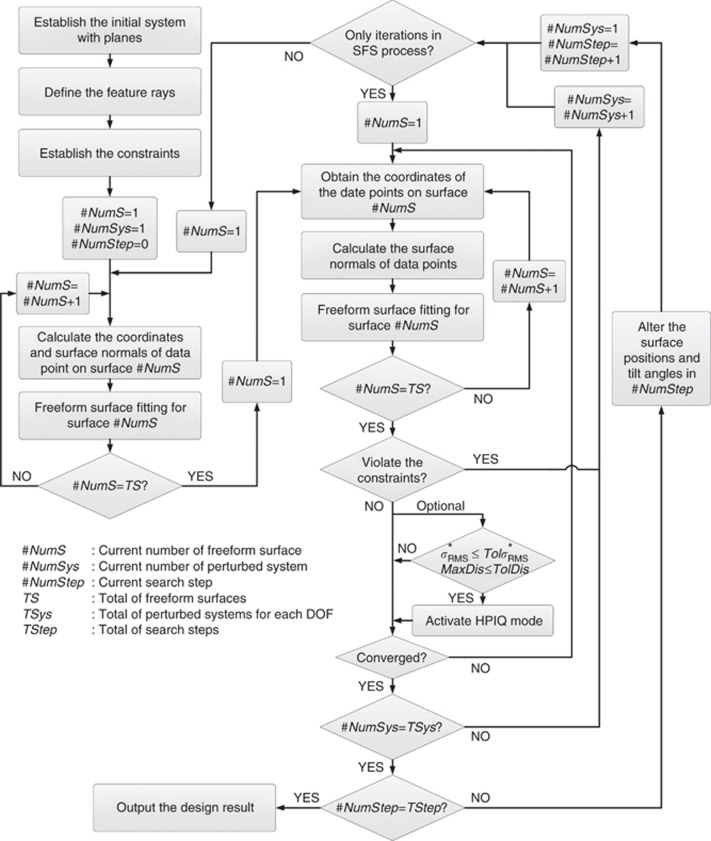
Flowchart of the design process.

An optional iteration mode called the high priority for image quality (HPIQ) mode is proposed here. In this mode, the target point **T**_***i***_ of each feature ray during the iteration process will no longer be the ideal image point of this field point. Instead, the target points of the feature rays for each field during the iteration process are the current intersections of its chief ray with the image plane. In this way, the feature rays of each field can be better focused to one point, thus improving the image quality. In this mode, another RMS deviation 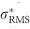 that only represents the image quality can be used to show the effect of the design process. The expression of 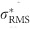 is similar to Equation (2). The only difference is that *σ*_*m*_ is replaced by 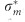, which is the distance between the actual image point for the *m*th feature light ray and its associated chief ray image point. Specifically, under the HPIQ mode, the iteration process of each perturbed system still uses the traditional mode at the beginning. During the iteration process, if 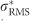 becomes smaller than a value *Tol*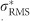 (which means that the image quality is not very bad) in one step during the iteration process and the maximum absolute distortion *MaxDis* does not exceed a predefined tolerance *TolDis*, the HPIQ mode will be activated at this time for the current perturbed system. From this time, the 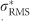 value is used to find the best solution after each search step in the following design process. Figure 4 shows the flowchart of the entire design process.

## Results and discussion

In this section, two examples are used to demonstrate the effectiveness of the design framework depicted in the previous section. Using our proposed automated design framework, we can obtain a high-performance freeform imaging system from simple initial planes automatically.

The first example is a freeform, off-axis, three-mirror system. This system works under the long-wave-infrared spectral band and has a 53 mm entrance pupil diameter. The F-number of the system is 1.798. The FOV of the system is 8° × 8°. The FOV is biased in vertical direction. The central field is (0°, −15°).

**Figure 5:**
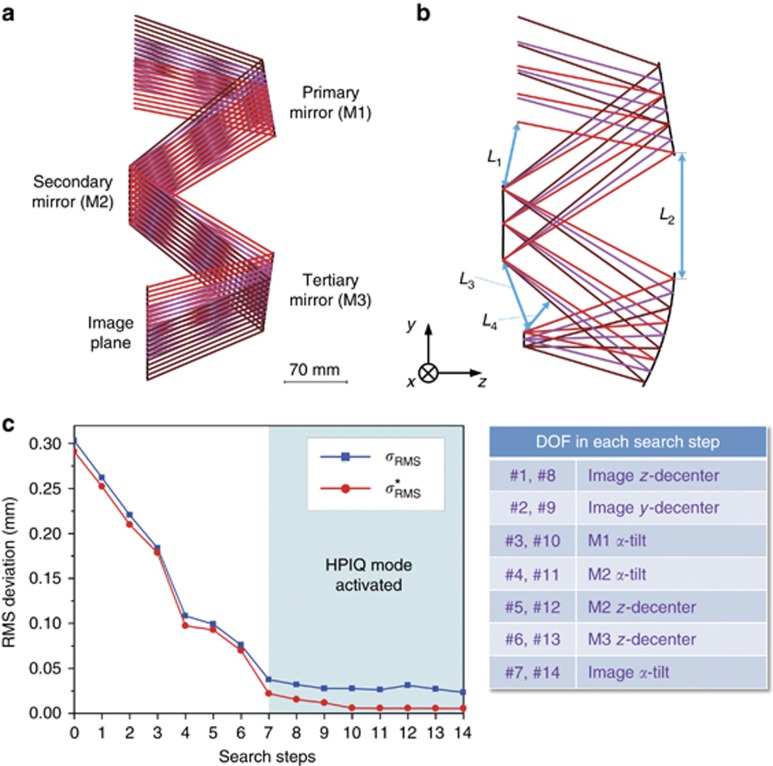
Design process of Example 1. (**a**) The initial system using planes. (**b**) Structure constraints. (**c**) The changes in RMS deviations with search steps.

An initial system using simple planes was first established as the input for the design, as shown in Figure 5a. The secondary mirror (M2) was the aperture stop. The feature rays were defined based on the polar ray grid. In the early stage of the design examples, we divide the aperture into 16 angles of different polar directions and we pick 7 pupil coordinates along each polar direction. We use 6 sample fields in half FOV during the design process: (0°, −11°), (0°, −15°), (0°, −19°), (4°, −11°), (4°, −15°), and (4°, −19°). Therefore, in total, *K*=6 × 16 × 7=672 feature rays were employed in the early stage of the design, whereas in the later stage, the total ray number was increased to *K'*=6 × 16 × 17=1632 (we pick 17 pupil coordinates along each polar direction). After defining the location of the image plane and the effective focal length of the system, the ideal target points for the feature rays were calculated. Then, some structure constraints were established. As shown in Figure 5b, the marked distances *L*_1_ to *L*_4_ have to be controlled to eliminate light obscuration or to avoid surface interference.

**Figure 6:**
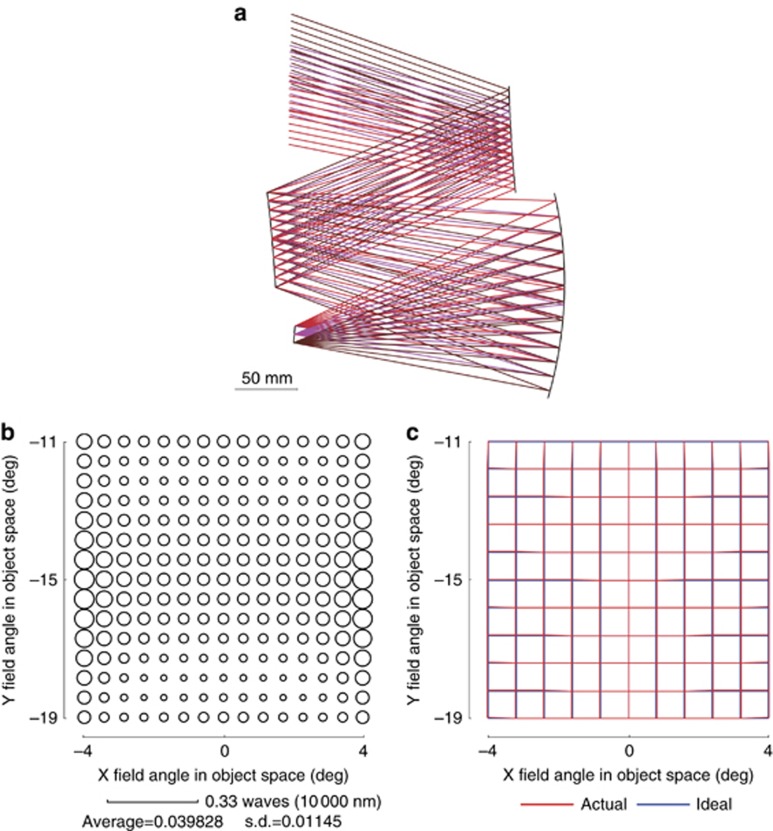
Final design result of Example 1. (**a**) Layout of the final design result. (**b**) RMS wavefront error of the final design result. (**c**) Distortion grid of the final design result.

Next, the automated design process started using the initial planar system as the design input. Negative feedback (*ε*=0.3) was used during iterations. The feature light rays were approximately redirected to their image points on the image plane in the system after the first CI process. However, this system was not yet ready for imaging because the image quality was far from the diffraction limit. The system designed by the first CI process was taken as the first base system (BaseS#1). In the following SFS steps, only the iteration process was repeated for each perturbed system. The construction process was not used. In total, 14 SFS steps were used in the process. All seven DOFs were used in sequence in steps 1–7. However, the design result after step 7 was not good. Therefore, the above search process was repeated using the same seven DOFs for another round to find a satisfactory result, starting from BaseS#8, which means the same seven DOFs were used again in sequence from search steps 8 to 14. The DOFs used for the SFS steps are listed in Figure 5c. The changes in *σ*_RMS_ and 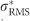 with the search steps are shown in Figure 5c. Here, the result of search step 0 in Figure 5c denotes the result of BaseS#1. The three mirrors used the fifth-order XY polynomial freeform surface in the early stage of the design and were then increased to sixth order in the later stage. A larger entrance pupil size was used during the early stage of the automated design process. From search step 8, the actual entrance pupil size was used and more feature rays (*K'*=1632) were sampled in the design. The HPIQ mode was activated during search steps 7–14. Figure 6a shows the final design result. Figure 6b shows the RMS wavefront error of the system. The average value was below 1/25 waves for 10 μm wavelength. The maximum absolute distortion was 60.8 μm. The distortion grid is shown in Figure 6c. These results show that the final system had a high performance. The total elapsed time for this design was 2.44 h. The final design data of Example 1 are given in Supplementary Information including the surface shape data and locations.

The initial planar system was extremely different from the final design result; thus, the traditional design process, which starts from a planar system, is very difficult. We tried to directly optimize this example in Code V starting from the same initial system with planes. To obtain a fair comparison, the structure constraints used in the optimization were the same as those used in the point-by-point design. The distortion was constrained using the real-ray-trace data of the chief rays. The variables were the curvatures, conic constants, all the coefficients of the sixth-order XY polynomials (including only even items of *x*) of the freeform surfaces, and the decenter and tilt values of all surfaces, including the image plane. However, the optimization process (both the standard and global optimization) could hardly continue and was unable to export useful and effective solutions. Nevertheless, experienced designers may be able to achieve good or even better design results in less time using software optimization with other design strategies when starting from an initial planar system. However, this approach requires considerable design experience. The point-by-point design framework proposed in this paper can realize the design of high-performance systems starting from simple planes. This strategy is different from the traditional design method based on the software optimization of surface coefficients, which start from a starting point with a certain optical power. The dependence on existing starting points is significantly reduced. In addition, the proposed design process reduces the amount of human effort required and does not rely on advanced design skills and experience. The time duration of the proposed method can be further reduced by using advanced search or optimization methods and advanced programming techniques.

**Figure 7:**
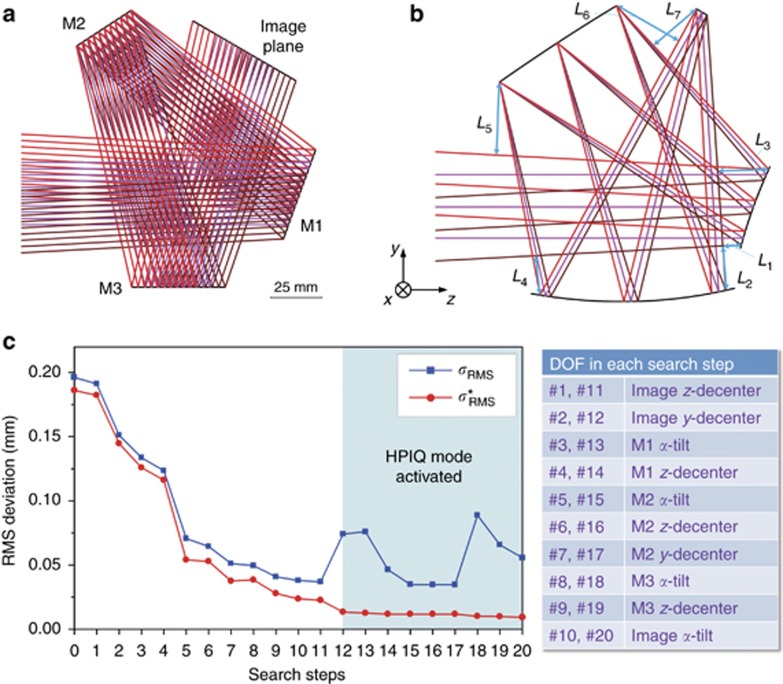
Design process of Example 2. (**a**) The initial system using planes. (**b**) Structure constraints. (**c**) The changes in RMS deviations with search steps.

The second example is a freeform reflective system with a special spherical package. A similar system was first designed by Fuerschbach *et al* using software optimization based on Nodal Aberration Theory^6^. This is a very good and compact design result. The FOV of the system is 8° × 6°. The system works under the long-wave-infrared spectral band with a 30 mm entrance pupil diameter and an F-number of 1.9. In our design process, we use 6 sample fields in half FOV: (0°, 3°), (0°, 0°), (0°, −3°), (4°, 3°), (4°, 0°), and (4°, −3°). First, an initial system using simple planes was established as the input for the design, as shown in Figure 7a. The secondary mirror (M2) was taken as the aperture stop. In total, *K*=6 × 16 × 7=672 feature rays defined by the polar ray grid were used in the early stage of the design. The method for sampling the rays was the same as that used in Example 1. The structure constraints used in this design are shown in Figure 7b. It should be noted that the constraints added in this example (as well as in Example 1) were structure constraints, which were used to eliminate the light obscuration. In fact, all kinds of constraints can be employed in the proposed automated design process.

**Figure 8:**
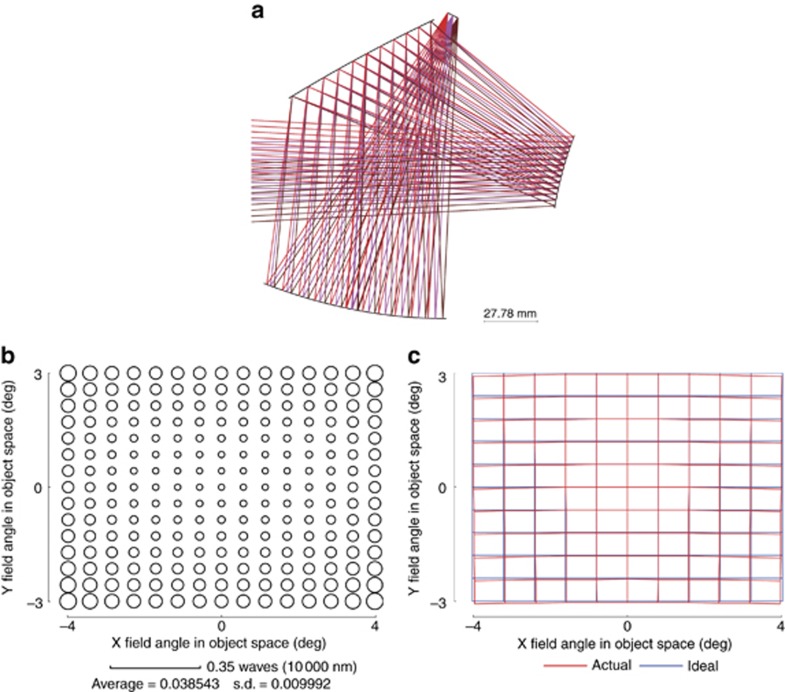
Final design result of Example 2. (**a**) Layout of the final design result. (**b**) RMS wavefront error of the final design result. (**c**) Distortion grid of the final design result.

Next, the automated design process was started. The iteration process used the negative feedback mode (*ε*=0.3). After the first CI process, the feature light rays were approximately redirected to their image points on the image plane in the system. This system was taken as the first base system (BaseS#1). Only the iteration process was repeated for each perturbed system during the SFS process. In total, 20 search steps were used in the SFS process. All the useful DOFs were employed once in search steps 1–10. Search steps 11–20 represented a second search round that involved the same DOFs as used in steps 1–10 to find a satisfactory result. Figure 7c shows the change in *σ*_RMS_ and 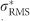 with the search steps as well as the list of DOFs used in the SFS steps. All the freeform surfaces used fourth-order XY polynomials in the entire design process. A larger entrance pupil size was used during the early stage of the automated design process. From search step 12, the actual entrance pupil size was used, and more feature rays (*K'*=6 × 16 × 17=1632) were sampled in the design. The HPIQ mode was activated during search steps 12–20. Figure 8a shows the final design result. Figure 8b shows the RMS wavefront error of the system. The average value was below 1/25 waves for 10 μm wavelength. The maximum absolute distortion was 96 μm. Figure 8c shows the distortion grid. These results show that the final system had a high performance. The total elapsed time for this design was 3.14 h. This time duration can be further reduced by using advanced search or optimization methods and advanced programming techniques. From the two design examples, we can also see that the locations of the initial planes are not exactly the same as those of the final design, but the whole folding geometry type is the same.

## Conclusions

We demonstrate that the automated design of high-performance freeform imaging systems can be achieved by using a novel point-by-point design framework. The freeform surfaces are not obtained via the traditional optimization of surface coefficients using optical design software. We instead use a point-by-point construction-iteration process. The entire design process is mostly automated and significantly reduces the amount of human effort. The designers only need to provide the initial planar system based on the configuration requirements or their prior knowledge. In this way, the dependence on existing starting points is also significantly reduced. Two design examples with high performance in the long-wave-infrared band are given to demonstrate the feasibility of the proposed design framework. This point-by-point design framework opens up new possibilities for automated optical design and may also be extended to optical design in many other areas.

To improve the design efficiency and generate better solutions, some other powerful techniques can be applied to the algorithm and the program, which are summarized as follows. (1) Advanced and fast optimization methods are needed. Currently, the positions and tilts of the freeform surfaces are altered via a single-freedom-search (SFS) process to achieve better design results. In fact, a search or optimization process using multiple degrees of freedom with smaller step sizes would be a better choice for generating good solutions. Therefore, advanced and fast optimization methods are needed. (2) Advanced programming techniques are needed. Currently, the automated algorithm is implemented in MATLAB (CODE V is also used as a design tool for ray tracing). To improve the efficiency, the program should be implemented in C or integrated directly into optical design software. (3) Parallel computing can be used to increase the speed of the design. (4) High-precision surface fitting methods are needed. The methods should be able to fit the coordinates and surface normals of data points onto all kinds of freeform surfaces as well as off-axis rotational symmetric surfaces. (5) Nodal aberration theory can be used to guide the point-by-point design in the later stage of the automated design process. (6) The point-by-point design framework can be used to design high-performance refractive systems when the correction of the chromatic aberration and the proper selection of materials are added to the algorithm. Using these techniques, we can explore the possibility of extending our design method for the fast and automated design of all types of high-performance optical systems.

## Author contributions

TY, GJ and JZ conceived the design method. TY wrote the programs and designed the systems. TY and JZ wrote the paper.
