# Supplementary material for: Automated design of freeform imaging systems
Source: Light Sci Appl. 2017 Oct 6;6(10):e17081–. doi: 10.1038/lsa.2017.81 (PMC6061905; doi:10.1038/lsa.2017.81)
Supplement: Supplementary Information [file lsa201781x1.doc]

**Supplementary information**

**Title: Automated design of freeform imaging systems**

**Authors**: Tong Yang, Guofan Jin, and Jun Zhu*

State Key Laboratory of Precision Measurement Technology and Instruments, Department of Precision Instrument, Tsinghua University, Beijing 100084, China

**Table S1.** Coefficients of the freeform surfaces in the final design of Example 1

| Term | M1 | M2 | M3 |
| --- | --- | --- | --- |
| *c* | 0.00077616 | −0.00094611 | −0.0031713 |
| *k* | 17.88333 | 13.63836 | 0.00509 |
| *A*_2_ | 6.81164E−04 | 2.46351E−03 | 1.94100E−04 |
| *A*_3_ | −4.02568E−05 | −4.64561E−05 | −2.27602E−05 |
| *A*_5_ | 2.86524E−05 | 8.19835E−05 | 2.72152E−05 |
| *A*_7_ | −1.75998E−06 | −4.14917E−06 | −2.34492E−07 |
| *A*_9_ | −6.61852E−07 | −2.46397E−06 | −5.16090E−08 |
| *A*_10_ | −2.42944E−09 | −1.67067E−08 | −1.02307E−09 |
| *A*_12_ | −2.45569E−09 | −3.38184E−08 | −1.21785E−09 |
| *A*_14_ | −2.85807E−09 | −1.67505E−08 | −5.76561E−10 |
| *A*_16_ | −1.12371E−11 | −1.53272E−10 | −1.58659E−12 |
| *A*_18_ | −2.15730E−11 | −2.54350E−10 | −2.25180E−12 |
| *A*_20_ | −1.03904E−11 | −1.02521E−10 | −2.18925E−13 |
| *A*_21_ | 4.37436E−14 | −4.68194E−13 | −1.55215E−14 |
| *A*_23_ | 1.58846E−13 | −1.92533E−12 | −3.77762E−14 |
| *A*_25_ | −5.84891E−13 | −2.18573E−12 | −2.96737E−14 |
| *A*_27_ | −4.82864E−14 | −6.76023E−13 | −8.16648E−15 |

Note: The expression of 6th order XY polynomial surface is

$\begin{matrix} z(x,y)=\frac{c(x^{2}+y^{2})}{1+\sqrt{1-(1+k)c^{2}(x^{2}+y^{2})}}+A_{2}y+A_{3}x^{2} +A_{5}y^{2}+A_{7}x^{2}y \\ +A_{9}y^{3}+A_{10}x^{4}+A_{12}x^{2}y^{2}+A_{14}y^{4}+A_{16}x^{4}y+A_{18}x^{2}y^{3} \\ +A_{20}y^{5}+A_{21}x^{6}+A_{23}x^{4}y^{2}+A_{25}x^{2}y^{4}+A_{27}y^{6} \end{matrix}$ (S1)

where *c* is the curvature of the surface, *k* is the conic constant, and *A_i_* is the coefficient of the *x-y* terms. As the optical system is symmetric about the *yOz* plane, only the even items of *x* are used.

**Table S2.** The decenter and tilt values of the surfaces in the final design of Example 1

| Surface | Global *y*-decenter (mm) | Global *z*-decenter (mm) | Global α-tilt (°) |
| --- | --- | --- | --- |
| M1 | 78.288 | 129.107 | 3.635 |
| M2 | 0.003 | −62.805 | 5.053 |
| M3 | −51.324 | 171.280 | −3.045 |
| Image plane | −75 | −45 | −5 |

Note: *y*-decenter and *z*-decenter are global decenter values of the surface vertices in *y* and *z* direction with respect to a global coordinate system in space. α-tilt values are the angles of the local surface *z*-axes with respect to the global *z*-axis. Here, α-tilt is the left-handed rotation angle about the +*x*-axis. The basic (local) coordinate system for each surface is a right-handed coordinate system with the surface oriented relative to the local z-axis. The system is symmetric about the *yOz* plane.
